# South African mental healthcare providers’ views about exercise for people with mental illness

**DOI:** 10.4102/sajpsychiatry.v30i0.2227

**Published:** 2024-04-30

**Authors:** Belinda S. Marais

**Affiliations:** 1 Department of Psychiatry, Faculty of Health Sciences, University of the Witwatersrand, Johannesburg, South Africa

**Keywords:** mental healthcare providers, views, exercise, physical activity, exercise prescription

## Abstract

**Background:**

People living with mental illness (PWMI) have significantly reduced life expectancy compared to the general population, yet mental healthcare providers (MHCPs) do not regularly prescribe exercise, despite its proven health benefits.

**Aim:**

This study aimed to evaluate South African MHCPs’ views regarding exercise for PWMI.

**Setting:**

A cross-sectional descriptive study conducted across five public sector specialised psychiatric units in Gauteng.

**Methods:**

Mental healthcare providers were surveyed using the Exercise in Mental Illness Questionnaire – Health Professionals Version.

**Results:**

Most participants were nurses (49.1%) and doctors (26.2%) and reported no prior formal training in exercise prescription (79.4%). The vast majority (up to 89.7%) agreed regarding the physical benefits of exercise, particularly cardiometabolic benefits. The most common barriers, as perceived by MHCPs, to exercise participation for PWMI were: stigma (56.5%) and medication side effects (56.5%). The majority (76.2%) reported prescribing exercise for PWMI at least ‘occasionally’. The method most frequently used was personal discussion (77.3%) and aerobic exercise was most frequently recommended (81.0%). Specific instructions regarding physical activity recommendations however were often not provided. Regarding MHCPs personal exercise habits, only a third (34%) met physical activity guidelines. Most MHCPs (92.1%) indicated an interest in further training regarding exercise for PWMI.

**Conclusion:**

Mental healthcare providers were supportive of exercise for PWMI. Strategies to address the stigma around mental illness and medication side-effects, to improve training regarding exercise prescription, as well as the exercise habits of MHCPs themselves, and collaboration with exercise professionals and organisations are recommended.

**Contribution:**

This study provides insight into the views of South African MHCPs regarding exercise for PWMI.

## Introduction

Exercise, or physical activity, has been proven to improve both physical and mental health and well-being.^1,2^ The World Health Organization (WHO) 2020 guidelines on physical activity and sedentary behaviour emphasise the importance of regular physical activity and the reduction of sedentary behaviours. The guidelines provide evidence-based recommendations regarding the types and frequency of physical activity required to confer significant health benefits and mitigate health risks. In addition, specific guidance is now included regarding subpopulations, such as pregnant and postpartum women and people living with chronic conditions and disabilities, including mental illness.^3,4^ The use of physical activity or exercise as a form of medical treatment for chronic disorders is not a new concept.^5^ ‘Exercise prescription’ refers to a specific plan of physical activities, given by the healthcare provider to the patient, for a specific therapeutic purpose.^5,6^

In the context of psychiatry, it has been well documented that people with mental illness (PWMI) have a reduced life expectancy of up to 20 years compared to the general population.^7^ A major contributing factor is believed to be because of increased rates of cardiovascular disease and metabolic syndrome among those with severe mental illnesses.^8,9^ Significantly lower physical activity levels and more sedentary behaviours have been reported among individuals with severe mental illness, highlighting the importance of exercise for PWMI, to improve cardiometabolic status and reduce morbidity and mortality.^10^ Furthermore, exercise can assist in the management of psychotropic medication-induced metabolic side effects.^11^ Exercise has also been shown to improve the symptoms of a variety of mental disorders, including severe mental illnesses.^11^ For example, in schizophrenia physical activity has been shown to be associated with improved global functioning, general psychopathology, positive, negative and cognitive symptoms, as well as quality of life.^4,12,13^ Similarly in bipolar disorder, physical activity has been shown to be associated with better health outcomes, in terms of less depressive symptoms and improvements in functioning and quality of life.^14^ Benefits of physical activity have also been found in children and adolescents, in terms of general mental health problems, as well as in those diagnosed with attention deficit hyperactivity disorder.^4,15^

Various barriers to exercise prescription and exercise participation have been reported in the literature, such as a lack of training in physical activity and exercise prescription, as well as the social stigma of mental illness.^16,17,18,19^ A lack of resources and organisational support has also been cited as an additional barrier.^16^ Healthcare professionals who themselves exercise regularly, as compared to those who do not, have been found to prescribe exercise more frequently and also reported less barriers to exercise prescription.^17^ In fact, regularly exercising professionals have been found to be 5–9 times more likely to prescribe exercise for PWMI and to provide specific details regarding physical activity recommendations.^18^

Regarding research findings related to exercise prescription practices, a survey of Ugandan mental healthcare providers (MHCPs), consisting of mostly nurses and a few occupational therapists, found that most MHCPs only ‘occasionally’ prescribed exercise. Most of the surveyed MHCPs further reported that they would like training regarding exercise prescription for PWMI, with topics of interest in particular being how to assess suitability for physical activity and also how to motivate patients to exercise.^20^ Another study conducted in the United States regarding exercise prescription practices for PWMI involving various subspecialities (including psychiatrists, psychologists, internal medicine and family medicine specialists) found that although many reported recommending exercise to their patients, only a minority actually provided specific exercise instructions and followed national physical activity guidelines in their recommendations.^18^ Similarly, in a study of psychiatric outpatients, most mental healthcare users reported that their MHCPs did not regularly speak to them about physical activity.^21^ It has also been found that rates at which exercise is prescribed to patients vary according to the type of mental illness, with it being recommended less frequently to those with schizophrenia, bipolar disorder and substance-related disorders, as compared to those with depression and anxiety.^16,18^ In the context of mental healthcare services in South Africa, little is known in this regard.

## Aim and objectives

This study aimed to evaluate the views of South African MHCPs, working at public sector specialised psychiatric units within Gauteng, regarding exercise for PWMI.

The study’s objectives were to determine:

- the knowledge and beliefs of MHCPs regarding the benefits of exercise for PWMI and its effectiveness in comparison to established treatments for mental illness
- mental healthcare providers’ perceived barriers to exercise participation for PWMI and MHCPs barriers to exercise prescription
- mental healthcare providers’ training, knowledge and confidence regarding exercise prescription, as well as their exercise prescription practices
- the personal exercise habits of MCHPs
- the training needs of MCHPs regarding exercise prescription.

## Research methods and design

### Study design and setting

This was a cross-sectional study conducted across specialised psychiatric units at various public sector hospitals in southern Gauteng, namely Chris Hani Baragwanath Academic Hospital, Charlotte Maxeke Johannesburg Academic Hospital, Helen Joseph Hospital, Sterkfontein Hospital and Tara Hospital. The MHCPs working at these sites consist of nurses, doctors, psychologists, occupational therapists, social workers and dieticians.

### Study population and sampling strategy

The study population consisted of MHCPs who form part of the psychiatric multidisciplinary team (MDT) working at the above-named hospitals. Convenience sampling was used. All MHCPs who provided informed consent to participate in the study, and who completed the study questionnaire, were included. Study questionnaires were distributed to MHCPs and, to ensure anonymity, were deposited upon completion into sealed boxes at the various sites.

### Data collection and instrument

Data collection took place over a 3-month period. The study questionnaire used to collect data from MHCPs was a modified version of the Exercise in Mental Illness Questionnaire – Health Professionals Version (EMIQ-HP). The EMIQ-HP is a self-administered questionnaire, developed by Stanton and colleagues in Australia to assess the knowledge, attitudes and behaviours of health professionals regarding exercise for PWMI. It consists of 68 questions and is divided into six parts: regarding exercise knowledge; exercise beliefs; exercise prescription behaviours; barriers to exercise prescription and exercise participation; exercise participation habits and demographics. Content validity and test-retest validity have been established for the EMIQ-HP.^22^ Permission to use the EMIQ-HP was obtained from the original author. For the purpose of this study, a few minor modifications were made to the EMIQ-HP to adapt it to suit the research context. The following changes were made: removed the question regarding bright light therapy, and questions regarding citizenship, highest level of education, year of graduation and preferred delivery mode for further training.

### Data analysis

Data were captured on Microsoft Excel and summarised using descriptive statistics – frequencies and percentages for categorical data and means and standard deviations for numerical data.

### Ethical considerations

Approval to conduct this study was obtained from the University of Witwatersrand Human Research Ethics Committee- Medical (reference no.: M221117). Institutional approval was obtained from the Research Committees and Chief Executive Officers of the various hospitals.

## Results

### Participants

**FIGURE 1:**
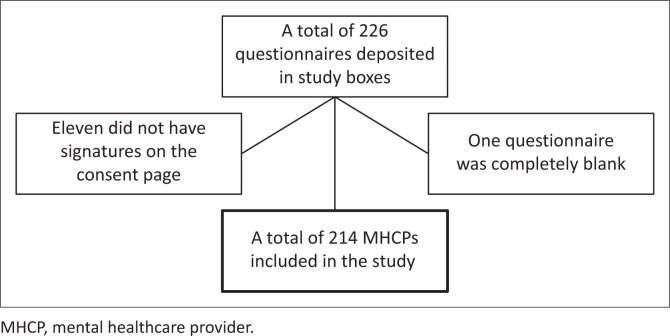
Flow diagram of mental healthcare providers participating in the study.

A total of 214 participants, across the five study sites, were included in the study (Figure 1). The characteristics of the MHCPs who participated in the study are shown in Table 1.

**TABLE 1:** Sociodemographic and professional characteristics of the mental healthcare providers participating in the study

MHCP sociodemographic and professional characteristics	*n*	%	Mean	s.d.
**Gender**				
Female	138	64.5	-	-
Male	68	31.8	-	-
Transgender	1	0.6	-	-
Did not answer	7	3.3	-	-
**Age (years)**	-	-	37.5	± 9.9
**Marital status**				
Single	127	59.3	-	-
Married	83	38.8	-	-
Did not answer	4	1.9	-	-
**Profession**				
Nurse	105	49.1	-	-
Doctor	56	26.2	-	-
Psychologist	18	8.4	-	-
Occupational therapist	17	7.9	-	-
Social worker	11	5.1	-	-
Dietician	4	1.9	-	-
Did not answer	3	1.4	-	-
**Years in profession**	-	-	11.0	± 8.5

MHCP, mental healthcare provider; s.d., standard deviation.

### Knowledge and beliefs

Knowledge and beliefs of the MHCPs regarding exercise for PWMI are shown in Table 2.

**TABLE 2:** Knowledge and beliefs of mental healthcare providers regarding exercise for people with mental illness.

Variable	Agree or strongly agree		Neither disagree nor agree		Disagree or strongly disagree		Did not answer	
	*n*	%	*n*	%	*n*	%	*n*	%
**Knowledge regarding the benefits of regular exercise**								
Maintenance of a healthy weight and prevention of certain chronic illnesses (e.g. type II diabetes, cardiovascular disease)	192	89.7	4	1.9	10	4.7	8	3.7
Improves total cholesterol	190	88.8	9	4.2	12	5.6	3	1.4
Improves blood pressure	189	88.3	10	4.7	11	5.1	4	1.9
Less likely to develop depression than those who do not exercise	159	74.3	36	16.8	16	7.5	3	1.4
Beneficial even if exercise is divided into shorter blocks of time (e.g. 10 min)	141	65.9	53	24.8	17	7.9	3	1.4
Reduces the risk of certain cancers (e.g. breast, prostate and colorectal)	117	54.7	68	31.8	24	11.2	5	2.3
**Beliefs regarding exercise for PWMI**								
Exercise benefits hospitalised PWMI in the same way as it does outpatients	189	88.3	10	4.7	11	5.1	4	1.9
PWMI know that exercise is good for their physical health	123	57.5	41	19.2	48	22.4	2	0.9
PWMI know that exercise is good for their mental health	78	36.4	54	25.2	79	36.9	3	1.4
PWMI do not exercise because they do not think they can	57	26.6	76	35.5	79	36.9	2	0.9
PWMI who are prescribed exercise will not adhere to it	55	25.7	104	48.6	48	22.4	7	3.3
The physical and mental health benefits of exercise for PWMI are not long-lasting	21	9.8	33	15.4	154	72.0	6	2.8

PWMI, people with mental illness.

Mental healthcare providers’ beliefs regarding the effectiveness of exercise in comparison to established treatment modalities for PWMI are shown in Table 3. Overall, between 76.2% and 92.1% of MHCPs viewed other treatment modalities to be of equal or greater value compared to exercise.

**TABLE 3:** Beliefs of mental healthcare providers regarding the effectiveness of exercise in comparison to established treatment modalities for people with mental illness.

Variable	Less effective than exercise		Of equal value to exercise		Better than exercise		Did not answer	
	*n*	%	*n*	%	*n*	%	*n*	%
**Beliefs regarding the effectiveness of exercise in comparison to other treatments**								
Medication	15	7.0	73	34.1	124	57.9	2	0.9
Cognitive behavioural therapy	21	9.8	70	32.7	119	55.6	4	1.9
Electroconvulsive therapy	45	21.0	48	22.4	115	53.7	6	2.8
Social support	14	6.5	93	43.5	104	48.6	3	1.4
Family therapy	21	9.8	91	42.5	99	46.3	3	1.4
Vocational rehabilitation	22	10.3	95	44.4	93	43.5	4	1.9
Social skills training	22	10.3	105	49.1	84	39.3	3	1.4

### Barriers to exercise participation and to exercise prescription

Mental healthcare providers’ responses to statements regarding barriers to exercise participation for PWMI, as well as MHCPs’ barriers to exercise prescription, are shown in Table 4.

**TABLE 4:** Perceived barriers to exercise participation for people with mental illness and barriers to exercise prescription by mental healthcare providers.

Variable	Agree or strongly agree		Neither disagree nor agree		Strongly disagree or disagree		Did not answer	
	*n*	%	*n*	%	*n*	%	*n*	%
**MHCPs perceived barriers to exercise participation for PWMI**								
Stigma around having a mental illness	121	56.5	30	14.0	60	28.0	3	1.4
Side effects from the medications	121	56.5	41	19.2	48	22.4	4	1.9
Uncertainty regarding what to do	109	50.9	50	23.4	52	24.3	3	1.4
Lack of confidence to do any exercise	105	49.1	43	20.1	62	29.0	4	1.9
Family or friends won’t exercise with them	95	44.4	62	29.0	54	25.2	3	1.4
No access to exercise equipment	83	38.8	39	18.2	88	41.1	4	1.9
Too many physical health problems	74	34.6	51	23.8	85	39.7	4	1.9
No safe place for PWMI to exercise	70	32.7	58	27.1	82	38.3	4	1.9
Too unwell to exercise	65	30.4	36	16.8	111	51.9	2	0.9
Feeling they are too fat to exercise	61	28.5	39	18.2	109	50.9	5	2.3
Fear of getting injured	61	28.5	54	25.2	95	44.4	4	1.9
Exercising is too time-consuming	55	25.7	26	12.1	130	60.7	3	1.4
**MHCPs barriers to exercise prescription for PWMI**								
Exercise might worsen their condition	4	1.9	16	7.5	187	87.4	7	3.3
Not interested in prescribing exercise	7	3.3	14	6.5	186	86.9	7	3.3
Not part of my job	21	9.8	37	17.3	153	71.5	3	1.4
PWMI are unable to participate in exercise due to their physical health	24	11.2	33	15.4	151	70.6	6	2.8
Concerns regarding PWMI getting injured while exercising	27	12.6	43	20.1	139	65.0	5	2.3
Workload is too excessive to include exercise prescription for PWMI	29	13.6	41	19.2	138	64.5	6	2.8
PWMI are unable to participate in exercise due to their mental health	35	16.4	52	24.3	119	55.6	8	3.7
PWMI will not adhere to an exercise programme	36	16.8	68	31.8	105	49.1	5	2.3
A lack of knowledge regarding exercise prescription for PWMI	66	30.8	49	22.9	95	44.4	4	1.9
Best delivered by an exercise professional	72	33.6	48	22.4	90	42.1	4	1.9

PWMI, people with mental illness; MHCP, mental healthcare provider.

### Exercise prescription practices

The majority (*n* = 170; 79.4%) of MHCPs participating in the study reported no prior formal training in exercise prescription. Of the remainder, 34 MHCPs (15.9%) reported having received training in exercise prescription, whereas 10 MHCPs (4.7%) did not respond to that question.

Regarding MHCPs’ knowledge of exercise prescription for PWMI, the following responses were obtained: poor or very poor (*n* = 32; 15.0%), average (*n* = 52; 24.3%), good or excellent (*n* = 49; 22.9%) and did not respond (*n* = 81; 37.9%). Responses regarding MHCP’s confidence to prescribe exercise to PWMI were: poor or very poor (*n* = 29; 13.6%), average (*n* = 53; 24.8%), good or excellent (*n* = 50; 23.4%) and did not respond (*n* = 82; 38.3%).

Three quarters (*n* = 163; 76.2%) of the MHCPs participating in the study reported that they prescribe exercise for PWMI: ‘occasionally’ (*n* = 70; 32.7%), ‘most of the time’ (*n* = 59; 27.6%) or ‘always’ (*n* = 34;15.9%). Of the remainder, 47 MHCPs (22.0%) reported that they never prescribe exercise for PWMI and four MHCPs (1.9%) did not respond.

Of the 163 MHCPs that reported prescribing exercise, the majority (*n* = 106; 65.0%) responded that they do not conduct a formal assessment to determine suitability for exercise, with the most frequent reason cited being a lack of knowledge or training, while a quarter (*n* = 42; 25.8%) responded that they do perform a formal assessment prior to prescribing exercise to PWMI. The assessments performed included conducting a physical examination, screening for physical disabilities, and considering age and medical comorbidities. Fifteen MHCPs (9.2%) did not answer the question regarding formal assessments.

Of the 163 exercise-prescribing MHCPs, the method most frequently used was personal discussion (*n* = 126; 77.3%). Other methods include: referral to an exercise professional or exercise facility, for example, a gym (*n* = 26; 16.0%), referral to community-based programmes (*n* = 24; 14.7%), ‘nothing specific’ (*n* = 20; 12.3%), brochures or pamphlets (*n* = 17; 10.4%) and ‘other’ methods (*n* = 16; 9.8%) such as incorporating it into the daily ward programme, group health discussions, occupational therapy groups, physical demonstrations and referring patients to online resources, videos or exercise applications (apps) on mobile devices.

Regarding the frequency of exercise, when prescribed for PWMI, the most common response was ‘as often as they feel they can’ (*n* = 47; 28.8%), followed by ‘most days of the week’ (*n* = 43; 26.4%), ‘every day’ (*n* = 39; 23.9%) or ‘once to twice a week’ (*n* = 35; 21.5%). The intensity of the exercise recommended by MHCPs prescribing exercise was most frequently ‘at a level that makes them feel good’ (*n* = 66; 40.5%) or ‘moderate intensity’ (*n* = 66; 40.5%), followed by ‘I do not suggest an intensity’ (*n* = 19; 11.7%) and ‘low intensity’ (*n* = 17; 10.4%). Almost half (*n* = 77; 47.2%) of the MHCPs who prescribe exercise recommended 30 min per session to PWMI, with other responses including 20 min (*n* = 32; 19.6%), ‘as long as they can’ (*n* = 30; 18.4%), 10 min (*n* = 24; 14.7%) and 60 min per session (*n* = 3; 1.8%).

The type of exercise most frequently recommended by MHCPs was aerobic exercise (*n* = 132; 81.0%), for example, walking, running or cycling. This was followed by team sports (*n* = 73; 44.8%), relaxation activities, for example, yoga (*n* = 63; 38.7%), weight or resistance training (*n* = 21; 12.9%), swimming (*n* = 20; 12.3%) and combat sports, for example, boxing and karate (*n* = 13; 8.0%).

### Personal exercise habits of mental healthcare providers

**FIGURE 2:**
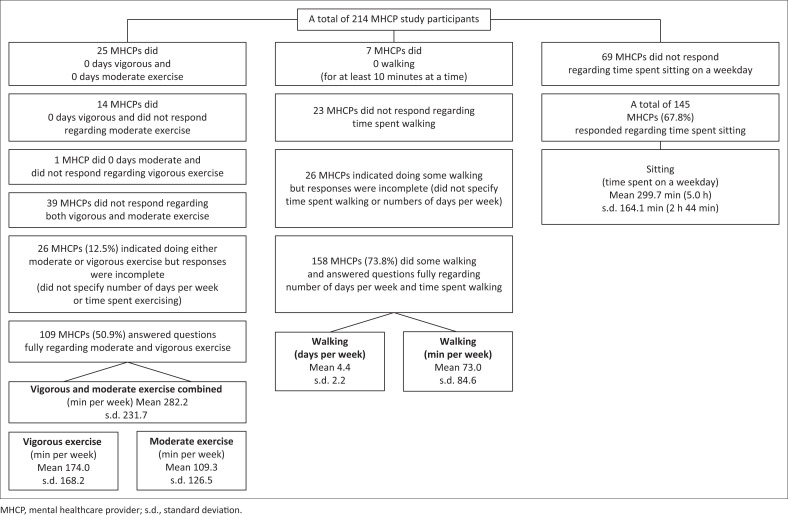
Mental healthcare providers’ own personal exercise habits and sedentary behaviours.

Mental healthcare providers were asked about their own personal exercise participation, specifically in the week preceding their completion of the study questionnaire. They were asked to provide details, in terms of number of days per week and time spent on those days, regarding participation in the following physical activities: vigorous physical activities (e.g. heavy lifting, aerobics and fast bicycling), moderate physical activities (e.g. bicycling at a regular pace, carrying light loads and playing tennis) and walking (for at least 10 min at a time). Mental healthcare providers were also asked about sedentary behaviours, in terms of time spent sitting per day. Responses are reflected in Figure 2. Overall only a third (*n* = 72; 33.6%) of MHCPs indicated participating in at least 150 min of moderate or vigorous exercise per week.

### Further training needs

The majority (*n* = 197; 92.1%) of MHCPs indicated that they would be interested, either ‘definitely’ (*n* = 141; 65.9%) or ‘possibly’ (*n* = 56; 26.2%), in further training for prescribing exercise for PWMI. Interest in further training according to the profession of MHCP was as follows: 53 doctors (94.6%), 98 nurses (93.3%), 16 occupational therapists (94.1%), 17 psychologists (94.4%), 10 social workers (90.9%), two dieticians (50%) and one MHCP who did not specify their profession. Of those who expressed interest in further training, preferred topics selected were: (1) how to get and maintain motivation in PWMI (*n* = 164; 83.2%); (2) how to assess the patient’s suitability for exercise (*n* = 159; 80.7%); (3) what type of exercise is best (*n* = 152; 77.2%) and (4) linking patients with community exercise programmes (*n* = 145; 73.6%).

## Discussion

### Key findings

#### Knowledge and beliefs

Overall MHCPs’ knowledge was good regarding the benefits of exercise, particularly in terms of weight, type II diabetes, cholesterol, hypertension, cardiovascular disease and depression, which is in keeping with the literature.^23^ The vast majority of MHCPs viewed other treatment modalities to be of equal or greater value compared to exercise, similar to the findings from the Ugandan study by Vancampfort et al. where this was the case in 74%–90% of the participants.^20^ Just over half of the MHCPs considered the following treatment modalities to be of greater benefit than exercise: medication, cognitive behavioural therapy and electroconvulsive therapy. This is in comparison to 77% and 65%, respectively, for medication and cognitive behavioural therapy (there was no question regarding electroconvulsive therapy, as that was not performed at their hospital) in the Vancampfort et al. study, where the authors observed a strong biomedical focus in the Ugandan healthcare system.^20^ The treatments most frequently viewed in this study, by just under half of the MHCPs, as being of equal value to exercise were: social skills training, vocational rehabilitation, social support and family therapy. In the Ugandan study, these treatment modalities were also most frequently viewed as being of equal value to exercise, but by a lesser percentage of MHCPs: social skills training, social support and family therapy in about a third of MHCPs and vocational rehabilitation in a quarter.^20^

#### Barriers to exercise participation

The most common barriers to exercise participation for PWMI, as perceived by MHCPs were: stigma around having a mental illness, medication side effects and uncertainty regarding what to do. This is in keeping with findings from the Ugandan study where the stigma and medication side effects were also the most commonly reported as barriers, although stigma was viewed as a much greater barrier, by 74% of their MHCPs.^20^ In an international survey of physical therapists regarding physical activity among people with schizophrenia specifically, patients’ lack of motivation was reported as the most common barrier by 45% of respondents, followed by lack of priority given to physical activity by other healthcare professionals in 28%.^24^

#### Barriers to exercise prescription

Encouragingly in this study, the vast majority of MHCPs *disagreed* that: they were not interested in prescribing exercise and that exercise prescription for PWMI is not part of their job, similar to the findings from the Ugandan study (87% and 77%, respectively).^20^ Furthermore, the majority of MHCPs *disagreed* that: exercise might worsen the condition of PWMI and that PWMI are unable to participate in exercise because of their physical health. However, a lack of knowledge regarding exercise prescription for PWMI, as well as exercise being best delivered by an exercise professional were identified as barriers to exercise prescription by a third of the MHCPs. In terms of this study, none of the study sites had exercise professionals, such as physiotherapists or physical therapists, as part of the psychiatric MDT. Internationally some countries have trained allied health professionals, referred to as physical therapists, or exercise physiologists, who facilitate the implementation of physical activity and lifestyle interventions for those with chronic illnesses, including mental illness. Additionally, the services of these professionals have been incorporated into mental healthcare settings, where they form part of the MDT involved in the treatment of PWMI. They are able to lead clinical exercise programmes, promote more physically active and less sedentary lifestyles through education, behaviour modification, motivational interviewing and other techniques, as well as assist in upskilling other MHCPs in exercise prescription.^25,26,27,28,29^ However, in most low-resource countries such physical activity professionals are not available, particularly not in mental healthcare, and those who are available are generally not trained in treating PWMI.^30^ Physiotherapists are allied health professionals who could potentially play a role within mental healthcare services in our setting and assist with physical activity interventions.^31,32^ However, a review of mental health policies in sub-Saharan Africa found that their importance in this context remains largely neglected.^31^ This is echoed not only by the dearth of research in sub-Saharan Africa regarding the role of physical activity in mental illness but also by the lack of priority that it has been given in mental health policies, where less than 10% even make reference to physical activity recommendations.^30^ A recently published review of the mental health education of physiotherapists found that physiotherapy students and physiotherapists have limited knowledge regarding mental illness, but despite this showed positive attitudes. Furthermore, the training of physiotherapists has been shown to lead to improved knowledge, attitudes and perceptions regarding mental health and PWMI. The recommendation by the authors of this review was therefore that the current South African physiotherapy curriculum be reviewed and improved to include sufficient mental health content so that ultimately physiotherapists may be better equipped to offer services to PWMI.^32^

#### Exercise prescription practices

The majority of MHCPs reported that they ‘prescribe exercise’ for PWMI at least ‘occasionally’. The method most frequently used was personal discussion. A much smaller proportion of MHCPs reported referring PWMI to an exercise professional *or* an exercise facility such as a gym (the study questionnaire did not specify which, but it is speculated by the author that these referrals were most likely to an exercise facility, such as a gym, rather than an exercise professional, as the study sites had limited access to such professionals and such referrals are not currently common practice in these settings.) The type of exercise most frequently recommended was aerobic exercise, whereas strength or resistance training was recommended by only a minority of the MHCPs. The WHO recommends that individuals perform aerobic physical activity, at a moderate to vigorous intensity, for at least 150 min per week, as well as muscle-strengthening exercises at least twice a week.^3,4^ ‘Exercise prescription’ refers to a specific exercise plan, which is given to an individual with a specific purpose and includes details regarding the type of exercise or physical activity, the intensity, the frequency and duration, as well as any necessary precautions. In other words, the term exercise prescription refers to more than simply advising the patient to ‘do some exercise’.^5,6^ In this study, the frequency of exercise that was most commonly recommended to PWMI was ‘as often as they feel they can’, followed by ‘most days of the week’. In terms of intensity, just over half of the MHCPs recommended either ‘at a level that makes them feel good’ or ‘I do not suggest an intensity’. These findings suggest that most MHCPs do not actually ‘prescribe exercise’ according to the true meaning of the term, in keeping with the results from other research.^18^ This also correlates with one of the top barriers to exercise participation by PWMI, as perceived by MHCP, found in this study: uncertainty regarding what to do. In terms of exercise, the uncertainty of PWMI regarding what to do and how to do it might therefore reflect the uncertainty of MHCPs themselves in terms of what exactly they should be advising to their patients.

#### Personal exercise habits of mental healthcare providers

Only a third (33.6%) of the MHCPs indicated personal participation in at least 150 min of moderate-to-vigorous exercise per week, as recommended by the WHO. This is not only a concerningly low proportion, but also less than what has been found among other MHCPs. In a 2019 survey of Dutch MHCPs, it was found that despite positive attitudes regarding physical health and exercise, less than half (43%) of the MHCPs met physical activity guidelines. Female gender, junior level of staff (intern/resident) and higher stress levels were factors found to negatively impact physical activity. It was further found that MHCPs were more likely to refer their patients for physical activity interventions if they themselves met physical activity guidelines.^23^ Levels of physical activity by South African MHCPs specifically could not be found in the literature. However, studies have been conducted in South Africa regarding the exercise participation of healthcare workers (HCWs) in general. A study performed on HCWs at a public tertiary hospital in Pretoria found a high prevalence of physical inactivity and obesity, among HCWs, while another study carried out in a private hospital in Johannesburg found that 39.6% and 48.8% of HCWs participated in the required amount of vigorous or moderate-intensity exercise, respectively. Furthermore, in both these studies, this was despite HCWs reporting good knowledge and attitudes regarding exercise. In other words, good knowledge by HCWs was not a significant predictor of physical activity.^33,34^ Thus, in addition to interventions to increase physical activity among PWMI, similar interventions specifically for MHCPs have also been recommended.^17,18^

#### Further training needs

The majority of MHCPs in this study reported no prior formal training in exercise prescription and most indicated that they would be interested in further training for prescribing exercise for PWMI. Of those who expressed interest in further training, topics selected were, in order of preference: (1) how to get and maintain motivation in PWMI, (2) how to assess patients’ suitability for exercise, (3) what type of exercise is best and (4) linking patients with community exercise programmes.

### Strengths and limitations

This was the first study conducted in South Africa on the views of MHCPs regarding exercise for PWMI, and it was conducted across multiple hospital sites in the public healthcare system. Findings of this study may however not be generalisable to other settings, such as community psychiatric clinics and the private mental healthcare sector. This was a descriptive cross-sectional study and no associations, causes or effects were explored. Furthermore, because of the sampling strategy employed in this study, the possibilities of social desirability bias, as well as non-response bias cannot be ruled out.

### Recommendations

The training of MHCPs regarding physical activity and exercise prescription for PWMI is recommended and with an additional focus on strategies to improve the physical activity of MHCPs themselves. Furthermore, strategies to address the barriers to exercise participation by PWMI identified in this study are recommended. Engagement with existing allied healthcare professionals available within our general healthcare services in the public sector, such as physiotherapists, is suggested to explore the prospect of services being extended to the mental healthcare context. Similarly, efforts to collaborate with other stakeholders to establish referral channels for PWMI to be able to engage in exercise programmes or access exercise facilities within the community should be made. The South African branch of the Exercise is Medicine (EIM) health initiative is one such potential stakeholder. Exercise is Medicine is a worldwide health initiative of the American College of Sports Medicine, aimed at making physical activity a standard component of the prevention and treatment of many chronic conditions and connecting healthcare providers with evidence-based exercise programmes and qualified exercise professionals for their patients.^35^ Together with the support of the South African Sports Medicine Association, a South African branch of EIM has been established.^36^ Research to investigate whether improved training and access to services translates into improved physical activity of PWMI would also be warranted, as would research into the mental and physical benefits, and cost-benefit analysis, of such interventions within the South African setting.

## Conclusion

Overall, MHCPs were knowledgeable regarding the benefits of exercise and supportive of exercise for PWMI. Stigma and medication side effects were the most common barriers to exercise for PWMI. Although the majority reported at least occasionally ‘prescribing’ exercise for PWMI, specific instructions, such as the frequency and intensity of exercise, were often not provided. Personal exercise habits of MHCPs were poor. Nonetheless, most of the MHCPs indicated that they would be interested in further training. Therefore, strategies to address the stigma around mental illness and medication side effects, to improve training regarding exercise prescription, as well as the exercise habits of MHCPs themselves, and collaboration with exercise professionals and organisations are recommended.
